# Hawley retainer and lichenoid reaction: a rare case report

**DOI:** 10.1186/s12903-019-0949-4

**Published:** 2019-11-20

**Authors:** Mahmoud Ahmed Elhadad, Yasmine Gaweesh

**Affiliations:** Oral Medicine and Periodontology Department, Faculty of Dentistry, Alexandria University, Alexandria, Egypt

**Keywords:** Oral lichenoid reaction, Lichen planus, Hawley retainer

## Abstract

**Background:**

Oral lichenoid reaction (OLR) is a type IV cell-mediated immune response in the oral cavity. There is an established relationship between various dental materials and OLR, but few cases reports reported the occurrence of a lichenoid reaction in association with the use of a Hawley retainer.

**Case presentation:**

A female patient (twenty years of age) has been complaining of a reddish painful area on the tongue, which started one year ago and has been increasing in size over time. The patient completed orthodontic treatment two years ago and has been using a Hawley retainer for orthodontic retention since then. After performing histological analysis and patch test, the lesion was diagnosed as a lichenoid reaction to the Hawley retainer. Topical corticosteroids were prescribed, and the patient was asked to stop using the retainer and followed for six months.

**Conclusions:**

It is difficult to diagnose lichenoid lesions and even more challenging to differentiate between OLP and OLR, therefore it is essential to do a full intraoral and extraoral examination. OLL can occur in association with Hawley retainer, which we believe could be because it is made of an acrylic based material. Generally, OLL resolves after removal of the cause.

## Background

Oral lichenoid reaction (OLR) or oral lichenoid lesion (OLL) is a condition, which is similar clinically and histologically to oral lichen planus (OLP). This term is used to describe eruptions of the oral cavity having an identifiable etiology [1].

The term OLR was first proposed by Finne et al. [2] in 1982 to designate clinically indistinguishable lesions of OLP. Furthermore, Laine et al. [3] in 1997, correlated OLL to contact allergy triggered by various dental materials.

Clinically, OLR may appear in any of the forms of OLP, namely reticular, plaque-like, erythematous, erosive, bullous, or ulcerative form [3] . Moreover, histologically, OLR and OLP are indistinguishable from one another. The main histological features of both lesions are damage of the basal keratinocytes and Inflammatory cell infiltration of the lamina propria that could extend into the epithelium [4].

When the previously mentioned clinical and histological features are present in a unilateral distribution in the oral cavity, OLL is the most probable diagnosis. Furthermore, the presence of dysplastic changes on the histological examination favors the diagnosis of OLL [5].

The following case-report presents full clinical and histological analyses of an oral soft tissue contact reaction to acrylic resin-based Hawley retainer in the form of an erosive area on the left and a keratotic area on the right dorsolateral surface of the tongue.

## Case presentation

**Fig. 1:**
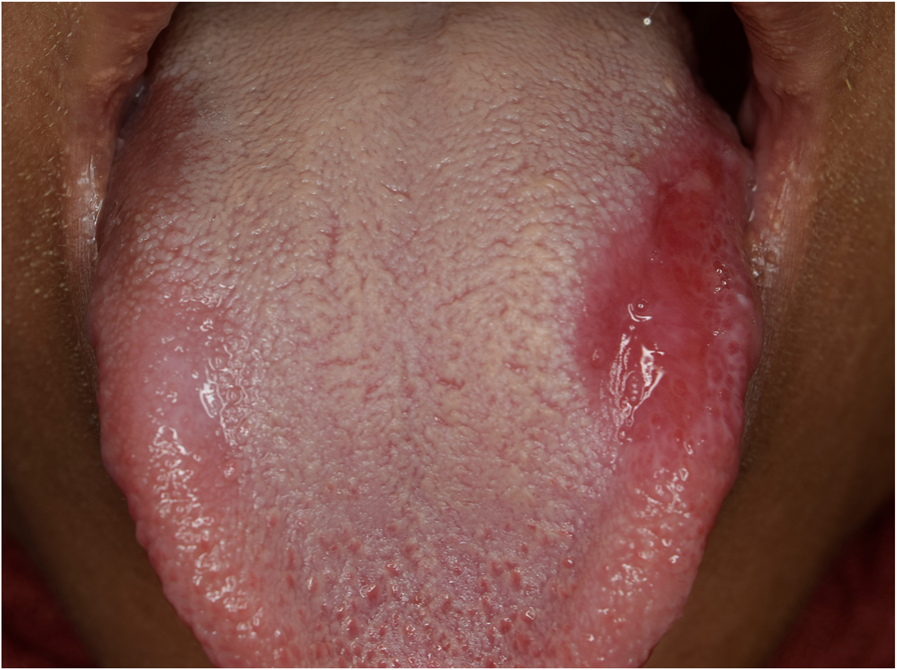
A photograph of the tongue showing fiery red lesion on the left side and white keratotic lesion on the right side

**Fig. 2:**
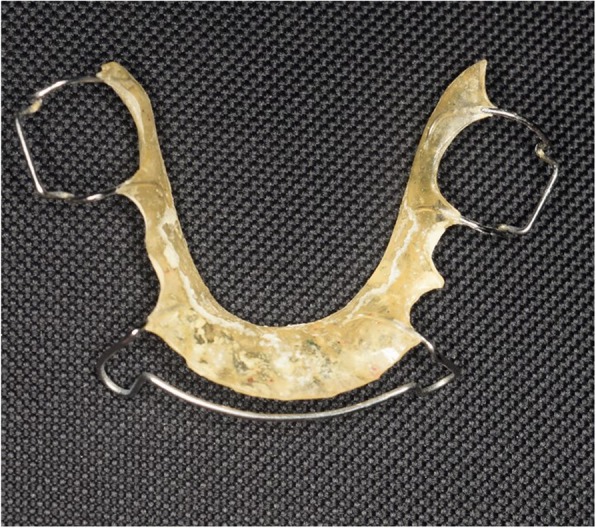
A photograph showing the acrylic-based Hawley retainer for the lower arch used by the patient

A twenty-year-old female presented to the oral medicine clinic at the Faculty of Dentistry, Alexandria University. The patient complained of a reddish painful area on the tongue, that started one year ago and has been increasing in size over time (Fig. 1). The patient reported completing an orthodontic treatment two years ago after which she has been using a Hawley retainer for orthodontic retention (Fig. 2).

**Fig. 3:**
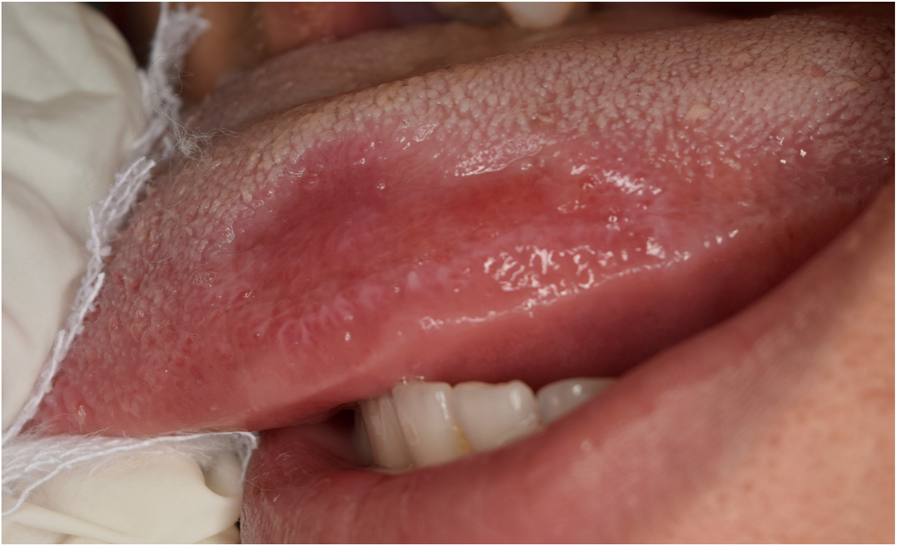
A photograph of the left dorsolateral side of the tongue showing a 2*3 cm erosive lesion surrounded by a keratotic line

**Fig. 4:**
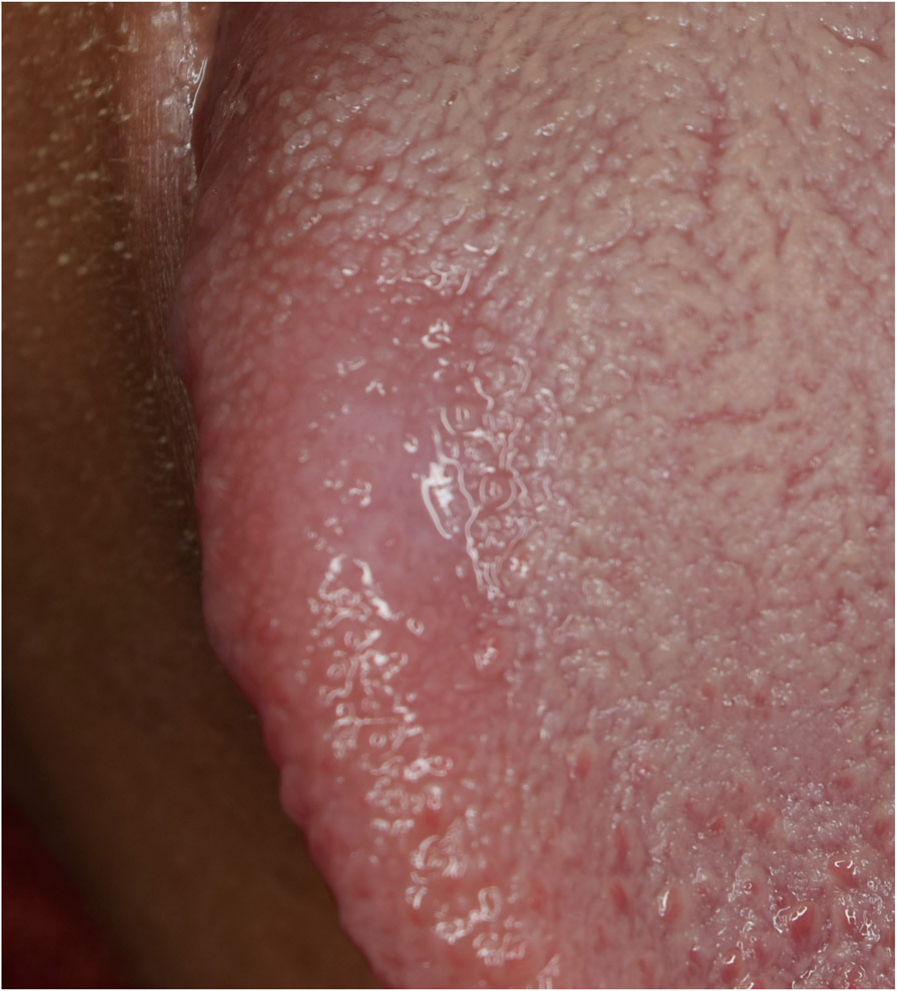
A photograph of the right dorsolateral surface of the tongue showing a one cm circumferential white keratotic lesion

No specific findings were found on medical history taking and extraoral examination. Intraoral examination revealed a reddish patch on the left dorsolateral surface of the tongue surrounded by whitish lines. The lesion measured 2 × 3 cm, had normal consistency, smooth surface texture, and normal surrounding tissues (Fig. 3). Another lesion in the form of a white keratotic plaque on the right dorsolateral surface of the tongue was found upon clinical examination. It was of 1 cm in size with normal consistency and normal surrounding tissues (Fig. 4).

The initial differential diagnoses included erythroplakia (because of the fiery red color), lichenoid contact reaction (because of the Hawley retainer), and geographic tongue (because of the location and age). An incisional biopsy was taken and subjected to histopathological examination to aid in reaching a conclusive diagnosis.

### Histopathologically

**Fig. 5:**
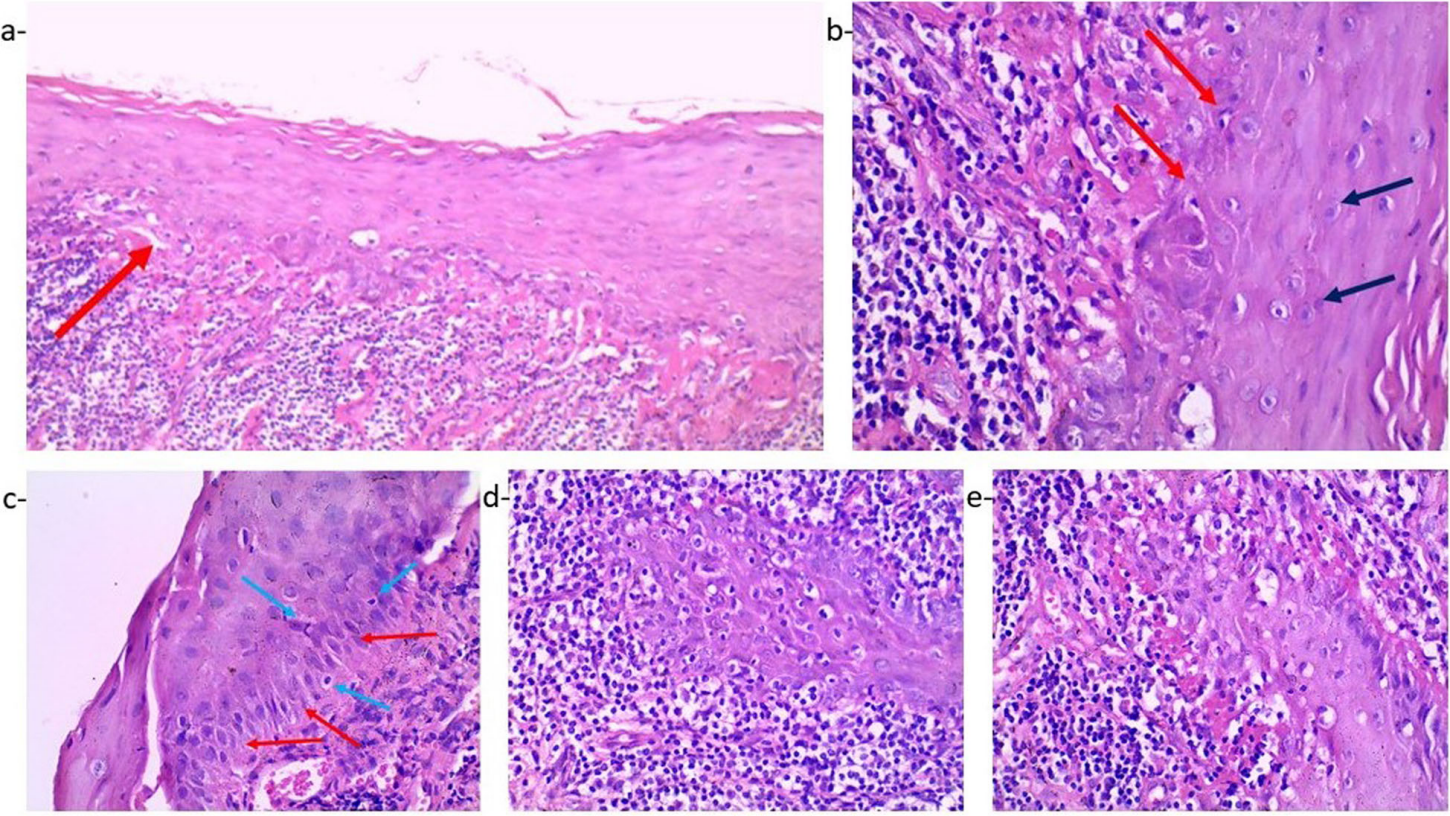
**a**: Microscopic examination of the lesion at (× 200) showing lichen planus with subepithelial lymphocytic infiltration and basal cell degeneration (red arrow). **b**: Higher magnification at (× 400) of the previous photomicrograph revealing the basal cell degeneration (red arrows) and prominent nucleoli (black arrows). **c**: Higher magnification at (× 400) of the epithelium showing different dysplastic criteria basilar hyperplasia (red arrows), apoptotic nuclei (blue arrows) as well as hyperchromatism and pleomorphism. **d**: Another higher magnification at (× 400) of the epithelium showing drop shaped rete pegs. Loss of the basal cells and subepithelial lymphocytic band. **e**: Higher magnification at (× 400) confirming the Loss of the basal cells and the existence of subepithelial lymphocytic band

The soft tissue section showed keratinized stratified squamous epithelium of variable thickness. Atrophic areas were predominantly present, other areas showed hyperplasia or epithelial proliferation in the underlying lamina propria. Degeneration of the basal epithelial cells and the basement membrane was evident. There was a dense, band-like lymphocytic infiltrate in the lamina propria that obscured the epithelial-connective tissue junction. Additionally, numerous dysplastic criteria such as hyperchromatism, pleomorphism, prominent nucleoli and mitotic figures were evident (Fig. 5).

### Management

**Fig. 6:**
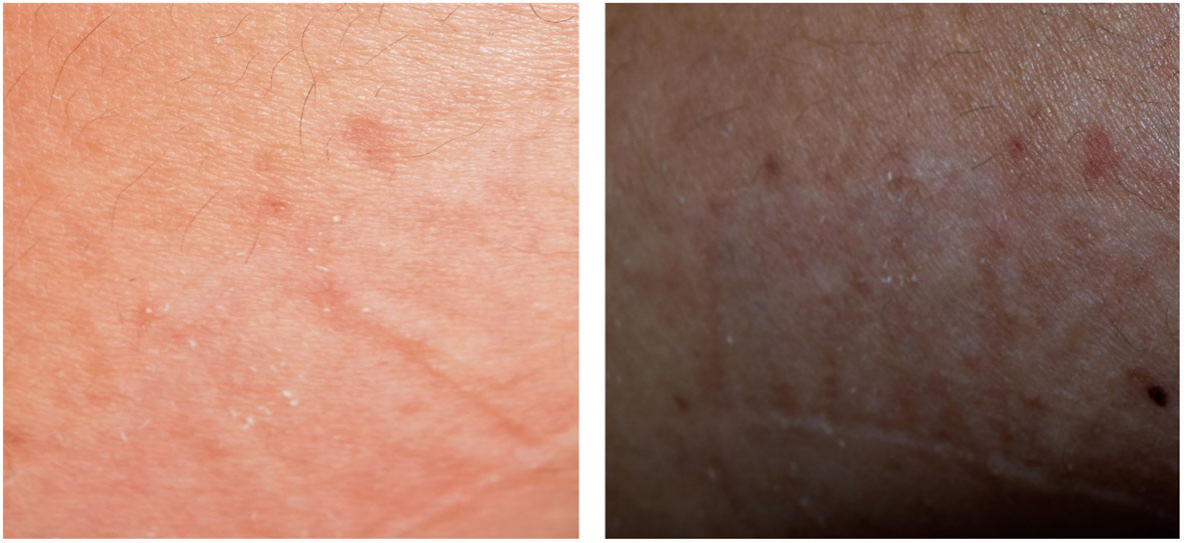
A photograph of the site of the hypersensitivity test showing delayed hypersensitivity reaction in the form of erythema, pigmentation and desquamation

Histopathological results suggested the diagnosis of OLR. In an attempt to confirm this diagnosis, we performed a patch test by applying grinded acrylic resin, similar to that used in the construction of the Hawley retainer, on the forearm for 72 h and instructed the patient to report any kind of discomfort. After 72 h, there was desquamation, erythema and pigmentation of the skin suggesting a positive patch test, which confirmed our diagnosis (Fig. 6).

**Fig. 7:**
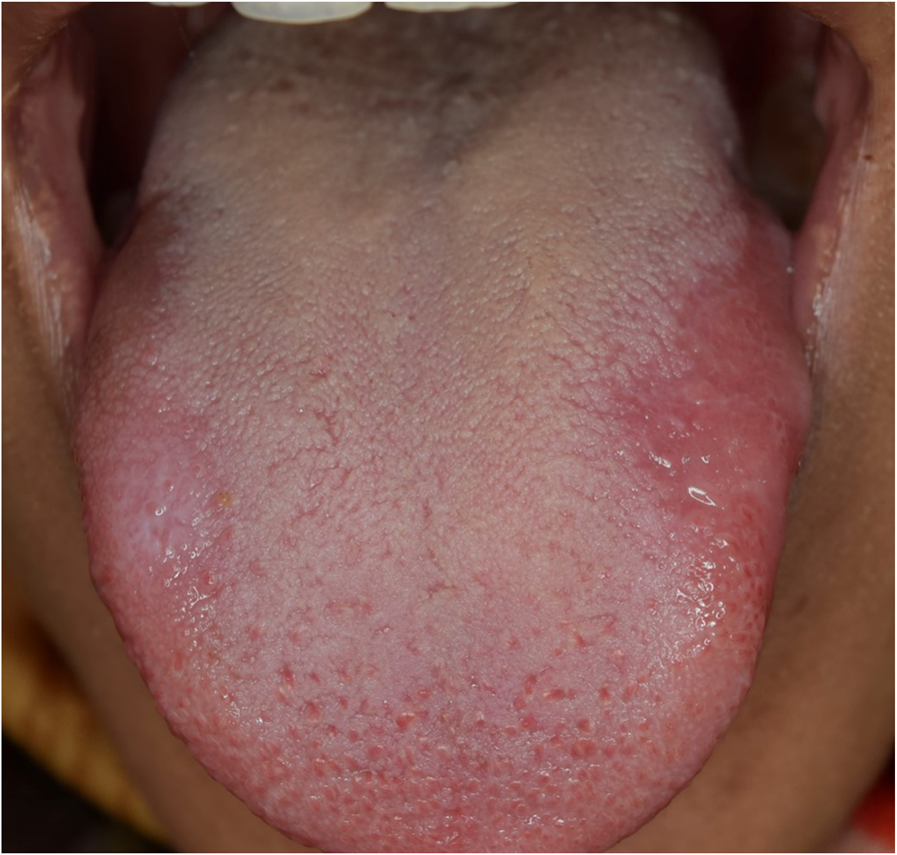
A photograph of the tongue demonstrating partial healing after six months with depapillation at the original site

We instructed the patient to discontinue using the Hawley retainer, replaced it by a vacuum retainer, and prescribed topical corticosteroids to decrease patient’s discomfort. Three weeks later, partial resolution of the lesion was evident. We followed the patient for six months with no signs of lesions recurrence (Fig. 7).

## Discussion and conclusion

In the presented case a definitive diagnosis was difficult to establish, therefore multiple diagnostic tools were implemented, namely clinical examination, histopathological analysis, and patch testing, which is useful whenever a dental material allergy is suspected [6].

Upon clinical examination, three entities were included in the differential diagnosis; erythroplakia, atrophic LP, and OLR, none of which could be excluded outright based solely on clinical appearance. Using histopathological examination, erythroplakia was excluded as the histopathological features matching those of LP and OLR were evident. Subsequently, the patch test demonstrated a positive result making the diagnosis of OLR more probable.

According to the diagnostic criteria proposed by van der waal [7]; the presented case can be considered “clinically and histopathologically compatible with OLP” but “not typical OLP”. Clinically, this is due to the lack of bilateral and symmetric distribution while histopathologically this is due to the presence of dysplastic changes.

According to Shirasuna et al. [8], Dudhia et al. [5], and several other studies [6, 9–11]: dysplasia is a possible feature of OLR and consequently OLRs might be liable to malignant transformation. On the other hand, OLP pre-malignancy is debatable [12–15]. Moreover, even though OLP and OLR appear clinically similar, OLR is usually unilateral in distribution. According to Kamath et al. [15], OLR is more frequently seen in sites with high risk for malignancy like; the tongue, floor of the mouth, and mandibular lingual alveolar ridge. Based on all the above-mentioned evidence, the most probable diagnosis for the presented case was OLR provoked by the acrylic resin material.

Rashid et al. [16] reported that acrylic based dental materials can lead to contact allergy manifesting as OLR. OLR should resolve after removal of acrylic based material and OLL would mostly involve contact sites; most frequently, lateral borders of the tongue, labial or buccal mucosa, and vestibular areas [5, 7, 13].

Numerous cases of lichenoid reactions or allergy due to self-curing resin were reported, however, lichenoid reactions linked to Hawley retainers were found to be extremely few. Case reports reporting allergic reactions to self-curing resin reported similar clinical presentations as most of them reported unilateral lesions, swelling and redness of the contact area [16, 17].

Tatiana et al. [18] reported an orthodontic case with allergy due to auto-polymerizing acrylic resin with a clinical presentation of a hypersensitivity reaction in the palate after using an orthodontic retainer. On the other hand, this did not occur when the residual monomer was analyzed with gas chromatography and was not above the international standards.

Furthermore, Alferdo et al. [19] reported a case of allergic reaction in a 33-year-old male. The patient reported discomfort and pain caused by an erythematous lesion located on the free and attached gingiva at the upper left first premolar site after the placement of an acrylic resin temporary restoration. Biopsy revealed a chronic inflammatory process. Notably, after cementation of the final crown, the inflammatory signs and symptoms disappeared.

Hawely retainer is the gold standard appliance for fixation and retention after orthodontic treatment. In addition, Hawely retainers are more favorable and more commonly used than vacuum retainers [20].

The presented case had some limitations. Most importantly, the patch test wasn’t performed using commercially available kits which would have given more precise results [21, 22]. Also, the use of topical corticosteroids in tandem with discontinuation of the use of the retainer confused the effect of each separately.

In conclusion, acrylic resin-based Hawley retainers should be used with caution watching out for possible similar adverse reactions. Further studies are required to explain and emphasize the existence of Hawley retainer-associated OLR in orthodontic patients. Establishing the possibility of such an association would necessitate the performance of a patch test for patients receiving this appliance after orthodontic treatment termination.

## Data Availability

All data generated or analyzed during this study are included in this published article.

## Abbreviations

OLDR: Oral lichenoid drug reaction
OLL: oral lichenoid lesion
OLP: oral lichen planus
OLR: oral lichenoid reaction
